# Longitudinal Assessment of Verbal Learning and Memory in Amnestic Mild Cognitive Impairment: Practice Effects and Meaningful Changes

**DOI:** 10.3389/fpsyg.2017.01231

**Published:** 2017-07-20

**Authors:** María Campos-Magdaleno, David Facal, Cristina Lojo-Seoane, Arturo X. Pereiro, Onésimo Juncos-Rabadán

**Affiliations:** Department of Developmental and Educational Psychology, University of Santiago de Compostela Santiago de Compostela, Spain

**Keywords:** California verbal learning test, subjective memory complaints, longitudinal design, standardized regression based methods, repeated assessments

## Abstract

**Objectives:** To identify learning effects and meaningful changes in amnestic mild cognitive impairment (aMCI) at a follow-up assessment.

**Method:** The Spanish version of the California Verbal Learning Test (CVLT) was administered to a sample of 274 adults of age over 50 years with subjective memory complains (SMC), including single and multiple domain aMCI groups and participants with SMC but without cognitive impairment (SMC group). The Wilcoxon test was used to compare results at baseline and after 18 months in short and long recall, and standardized regression-based (SRB) methods were used to study meaningful changes.

**Results:** Scores were significantly higher at follow-up for short and long-delayed recall in all groups indicating generalized practice effect. SRB scores indicated a significant decline in recall in a higher proportion of participants with aMCI than in SMC group.

**Discussion:** Patients with multiple and single domain aMCI benefit from practice in a verbal learning memory test. The SRB approach revealed a higher incidence of meaningful decline in short and long-delay recall and recognition in the aMCI groups than in the SMC group. Specifically, compared to SMC participants, single-domain aMCI individuals declined in a higher proportion in all measures, and multiple-domain aMCI individuals in long delay free recall.

## Introduction

The ability to learn and retain new information is one of the most age-sensitive cognitive domains (Kramer et al., 2003; Dixon et al., 2004; Josefsson et al., 2012) and tends to deteriorate more rapidly in patients with amnestic Mild Cognitive Impairment (aMCI) who subsequently develop Alzheimer Disease (AD; Albert et al., 2011). One of the tools most widely used to assess verbal learning and memory is the California Verbal Learning Test (CVLT; Delis et al., 1987), based on word lists learning.

When longitudinal designs are implemented to study the temporal course of cognitive decline, the measurement and analysis of change must be carefully considered. Practice effects have to be taken into account to avoid underestimating decline when measuring instruments are used repeatedly (Rabbit et al., 2004; Salthouse et al., 2004; Knight et al., 2007). The cognitive decline might also be influenced by the time elapsed between assessments, which can also modulate the magnitude of the practice effect. The practice effect is readily observed, mainly when intervals are very short (i.e., 1 week or 1 month), as in studies carried out by Krenk et al. (2012) and Woods et al. (2006). Calamia et al. (2012) noted a negative effect of the length of test-retest interval, indicating that longer retest intervals are associated with lower estimated score gains at retesting.

There is evidence for the universality of the practice effect in verbal learning and memory, and its effects have been observed in healthy older adults (Woods et al., 2006; Machulda et al., 2013)—even in very old adults (Balasubramanian et al., 2012)—and in patients suffering from MCI and from dementia (Calamia et al., 2012; Machulda et al., 2013). In a recent longitudinal study, the Mayo Clinic research group found that cognitively normal participants who developed incident MCI or dementia showed an initial practice effect in memory and that those who developed incident MCI or dementia at visit 3 or later (or at least 30 months following baseline) showed the most notable practice effects, suggesting that even in this group practice effects persist a few years before the onset of MCI/dementia (Machulda et al., 2013). In a meta-analyses, Calamia et al. (2012) showed a similar practice effect in MCI patients and in the non-clinical population, but a less notable effect in AD patients. The improvements in cognitive performance can be linked with implicit memory, which tends to be maintained through normal ageing (Swick and Knight, 1997) and AD (Knopman and Nissen, 1987; Bozoki et al., 2006) or to decrease slightly with increasing age (Howard and Howard, 1998), while declarative memory tends to deteriorate progressively.

In this context, the study of the natural history of the cognitive decline must consider the significance of change over the lifespan. Numerous methods are available for evaluating the significance of change, including the simple Reliable Change Index (RCI) method (RCI; Jacobson and Truax, 1991), the RCI with correction for practice effects (Chelune et al., 1993), a simple regression model that predicts follow-up from baseline scores (Speer and Greenbaum, 1995), and the standardized-regression-based (SRB) methods (McSweeny et al., 1993). The SRB method involve deriving a predicted follow-up score based on initial test performance in a control sample and including the regression scores at base line and other potential predictors (e.g., retest interval, age, education, etc.). With the exception of the simple RCI method, in which the practice effect is not controlled for, the accuracy of prediction of the other models is comparable and overall higher than that of the simple RCI (Temkin et al., 1999; Heaton et al., 2001; Frerichs and Tuokko, 2005).

Some previous studies have measured practice effects using healthy samples or MCI samples, but not MCI subtypes (Dixon et al., 2004; Duff et al., 2007, 2008; Knight et al., 2007; Krenk et al., 2012). Study of practice effects in the CVLT in different subtypes of aMCI may provide clinically useful information about variations in progression of memory impairment (Duff et al., 2008). The present study aimed to identify patterns of change of verbal learning and memory in people with subjective memory complains and with two subtypes of aMCI (single and multiple domain) which represent different degrees of severity along the continuum between normal aging and dementia (Brambati et al., 2009; Han et al., 2012). We also aimed to provide standardized change scores for the CVLT by using the SRB method to identify meaningful change in both subtypes of aMCI patients relative to participants with subjective memory complaints (SMC) but without cognitive impairment (SMC group). As practice effects may be enhanced by short intervals between assessments (Rabbit et al., 2004; Salthouse et al., 2004; Knight et al., 2007) and as practice effects may mask cognitive changes, we aimed to identify the pattern of meaningful change in verbal learning and memory during a period of around 18 months, which is a commonly used interval for follow-up assessments in MCI studies (Machulda et al., 2013).

## Methods

### Participants

Two hundred seventy-four adults over 50 years old (range 50–87; mean 66.53) who completed the cognitive and neuropsychological assessment at baseline (T1) and at the first follow-up (T2) where selected for this study from the sample of a larger longitudinal study on cognitive decline (Juncos-Rabadán et al., 2012). All participants were recruited from primary care health centers in Spain and referred to us by general practitioners. The inclusion criteria for the study were: (1) the presence of SMC, and (2) no prior diagnosis of MCI or dementia, clinical stroke, traumatic brain injury, motor-sensory defects, alcohol or drug abuse/dependence, or diagnosis of any neurological or psychiatric disease. Participants were classified into two aMCI subtypes, a multiple domain aMCI group (mda-MCI; *n* = 21) and a single domain aMCI group (sda-MCI; *n* = 46), and a reference group with no MCI but with Subjective Memory Complaints (SMC group; *n* = 207). Patients with non-amnestic MCI were excluded. The aMCI participants met the general criteria outlined by the National Institute on Aging-Alzheimer's Association (Albert et al., 2011): (a) informant-corroborated memory complaints assessed by the Subjective Memory Complaints Questionnaire (SMCQ; Benedet and Seisdedos, 1996; short version); (b) performance of 1.5 standard deviations (SD) below age norms on the of the Verbal Paired Associates-Immediate and Delayed subtests of the Wechsler Memory Scale-Third Edition (Wechsler, 1999, Spanish version); (c) no significant impact on activities of daily living assessed by the Lawton and Brody Index (Lawton and Brody, 1969); and (d) not demented according to the National Institute of Neurological and Communicative Disorders and the Alzheimer's Disease and Related Disorders Association and Diagnostic and Statistica Manual of Mental Disorders-fourth edition criteria.

The patients with mda-MCI also scored 1.5 SDs below age- and education-related norms on at least two cognitive subscales of the Spanish version of the Cambridge Cognitive Examination, CAMCOG-R (López-Pousa, 2003; Pereiro et al., 2015), which assesses deterioration in specific domains such as language, attention-calculation, praxis, perception, and executive functioning (Huppert et al., 1996; Cullum et al., 2000) and detects prodromal AD in mild cognitive impairment (Gallagher et al., 2010). The general cognitive functioning of this group was around 1.5 SDs below age- and education-related norms in the Spanish version of the Mini-Mental State Examination (Lobo et al., 1999). Patients with sda-MCI scored 1.5 SDs below age norms in the memory tests and maintained normal general cognition, as measured by the Mini-Mental State Examination and the CAMCOG-R subtests. Activities of daily living assessed by the Lawton and Brody index (Lawton and Brody, 1969) were intact or minimally affected. SMC group scored higher than the cut-off in memory, general cognitive functioning, and specific cognitive domain tests. All participants of the SMC group performed as cognitively normal adults according norms by age and years of education. All diagnoses were reached by consensus, at a special meeting of the research team and taking into account only the Wechsler Memory Scale-Third Edition scores for evaluating memory. The demographic and neuropsychological profiles of the participants are summarized in Table 1 that includes group comparisons (parametric, ANOVA, or not parametric, Kruskal–Wallis depending on the homogeneity of the variance tested with Levene procedure).

**Table 1:** Mean scores (and standard deviations) for demographic and neuropsychological variables obtained at Time 1 by the different study groups: single domain amnestic mild cognitive impairment (sda-MCI), multiple domain amnestic mild cognitive impairment (mda-MCI), and a group with subjective memory complaints without cognitive impairment (SMC).

**Variable**	**SMC (*N* = 207)**	**sda-MCI (*N* = 46)**	**mda-MCI (*N* = 21)**	***F*_(2, 271)_*χ^2^*_(2, 274)_**	**Groups comparison (Bonferroni)**
Gender	Men: 30%	Men: 54.3%	Men: 9.5%		
	Women: 70%	Women: 45.7%	Women: 90.5%		
Age	65.30 (8.87)	69.67 (8.75)	71.86 (8.43)	*F* = 8.76^***^	sda-MCI, mda-MCI > SMC
	Range: 50–87	Range: 52–87	Range:54–87		
Years of education	9.74 (4.47)	9.41 (4.12)	9.86 (3.92)	*F* = 0.12	
	Range: 1–22	Range: 2–20	Range: 3–18		
Retest interval	18.96 (4.54)	19.32 (6.32)	21.48 (8.96)	*χ^2^* = 1.01	
MMSE	28.20 (1.43)	27.46 (1.76)	23.33 (1.37)	*χ^2^* = 60.78^***^	mda-MCI < sda-MCI < SMC
Memory complaints (participant)	19.18 (4.38)	19.38 (4.47)	18.92 (3.37)	*F* = 0.46	
Memory complaints (informant)	15.52 (4.20)	16.82 (4.48)	16.75 (4.88)	*F* = 1.78	
Language	25.70 (2.40)	25.10 (2.64)	23.91 (1.83)	*F* = 12.57^***^	SMC, sda-MCI > mda-MCI
Attention-calculation	7.61 (1.54)	7.46 (1.60)	5.92 (2.61)	*χ^2^* = 19.83^***^	SMC, sda-MCI,> mda-MCI
Executive function	18.40 (4.13)	16.47 (3.88)	13.90 (3.19)	*F* = 14.53^***^	mda-MCI < sda-MCI < SMC
WMS-III (Immediate auditory memory)	98.91 (11.52)	85.91 (10.15)	82.10 (10.34)	*F* = 39.96^***^	sda-MCI, mda-MCI < SMC
WMS-III (Delayed auditory memory).	111.39 (14.30)	97.12 (12.74)	92.70 (12.88)	*F* = 30.07^***^	sda-MCI, mda-MCI < SMC
CCI	0.85 (0.88)	0.80 (0.90)	0.76 (0.76)	*F* = 0.16	
	Range: 0–3	Range: 0–3	Range: 0–2		

*Gender distribution is indicated with percents. MMSE, Mini-Mental State Examination; WMS-III, Wechsler Memory Scale-Third Edition; CCI, Charlson Comorbidity Index*.

*** *p < 0.0001*.

After the baseline assessment, the participants were informed that they would be called for a follow-up evaluation after about 18 months. For the purposes of this study, we selected all those participants who completed the Verbal Paired Associates-Immediate and Delayed subtests of the Wechsler Memory Scale-Third Edition at baseline, and who completed the Spanish version of the CVLT at baseline and at the follow-up assessment. Reason for not continuing in the study, and consequently not completing follow-up assessments, included motivation, morbidity, physical health, conversion to dementia and mortality (see Facal et al., 2016 for further information about attrition).

Written informed consent was obtained from all participants prior to the study, which met with the approval of the Research Ethics Committee of the Xunta de Galicia (Spain) and was conducted in accordance with the provisions of the Declaration of Helsinki, as revised in Seoul 2008.

### Procedure and materials

We used the Spanish version of the CVLT (Delis et al., 1987; Test de Aprendizaje Verbal de España-Complutense; Benedet and Alejandre, 1998), since the more recent revision of the test (CVLT-II) was not available in Spanish. This test has proven to have adequate reliability (odd pair correlation, 0.94; split-half correlation, 0.82) and validity (factorial structure explains 67% of the variance; Benedet and Alejandre, 1998, pp. 27–31). A list of 16 words (four from each of four semantic groups) was presented orally to the participants, who were required to recall the words immediately, after short and long delays and also with and without semantic cues. A recognition task was then administered. The test included the following measures to reflect learning and recall, also called primary variables: Total Trial 1–5, Short Delay Free Recall (SDFR), Short Delay Cued Recall (SDCR), Long Delay Free Recall (LDFR), and Long Delay Cued Recall (LDCR). It also included other variables that reflect processing of information and other cognitive processes such as Semantic Clustering and Serial Clustering, Region effects, Intrusions, and Perserverations (see Campos-Magdaleno et al., 2014, for a more detailed explanation of the test). We considered only the primary variables for the purposes of this work.

The CVLT was administered to all participants at T1 and T2 after a previously established interval of about 1 year and half (*M* = 19.21; *SD* = 5.33; Facal et al., 2014). No parallel version of the CVLT was used due to unavailability in Spanish during assessments.

### Statistical analysis

The first phase of analysis examined the extent of practice effects on each of the CVLT primary variables (raw scores). The skewness parameters revealed the homogeneity of distribution of the variables Total Trial 1–5, Short Delay Free Recall (SDFR) and Long Delay Free Recall (LDFR) and the no-homogeneity of the variables Short Delay Cued Recall (SDCR), and Long Delay Cued Recall (LDCR) for the SMC group. For the sda-MCI and mda-MCI all variables showed homogeneous distribution. Taking into account the non-homogeneity of two variables in the SMC groups and the small size of the two aMCI groups we decided the application of non-parametric analysis. The Wilcoxon signed-rank test (*z*) was performed to determine which variables changed significantly between T1 and T2, and Spearman's rho (ρ) was used to obtain correlation coefficients for assessing test-retest reliability. The magnitude of changes between assessments was measured with Cohen's *d* (Fritz et al., 2012).

In a second phase, the regression-based prediction of follow-up test scores was calculated using the SRB approach (McSweeny et al., 1993). This involves deriving a predicted follow-up score based on initial performance by a control sample. We took the SMC group as control or reference sample. The basic strategy of the regression analysis consists of determining the variables that might affect performance at follow-up in the SMC group. The variables entered in the regression were the baseline score in Total Trial 1–5, SDFR and LDFR (the CVLT variables that showed homogeneous distribution), age, education, and test-retest interval. The regression-based predicted scores of follow-up were then calculated according to the equation Y_p_ = β_1_X_1_ + β_2_X_2_ + β_3_X_3_ + β_4_X_4_ + C, where Y_p_ is the predicted follow-up score, β is the regression coefficients (slopes) for each of the entered variables, X_1_ is the observed baseline score, X_2_ is the age, X_3_ is the education, X_4_ is the test-retest interval, and C is a constant (intercept). The equation was developed by considering the SMC group as control population used to develop the change norms.

Once the predicted score was established, the standardized change score for each individual was calculated as follows: *z* change score = (Y_2_ – Y_p_)/S.E.E, where Y_2_ is the particular score in Tfor one individual, Y_p_ is the predicted follow-score for the SMC group, and S.E.E is the standard error of estimate in the regression equation. On obtaining the *z* change score, further calculation can provide individual levels of determination of change. Statistically reliable change may be based on change scores that exceeded a z score value of ±1.64 change score units (90% Confidence Interval, CI; McSweeny et al., 1993). Individuals for whom the *z* change score fell within the 90% CI were classified as cognitively “stable,” for that variable, whereas scores outside the CI were designated as having significantly “declined” or “improved” as appropriate.

Finally, we performed cross-tabs for all the CVLT measures to compare proportion of individuals in the three groups whose performance remained stable, declined or improved, using chi-square analyses to assess global significance, the Cramer's *V* to assess the intensity of the association and corrected standardized residuals (*CSR*, Haberman, 1973) to determine the specific associated categories. All analyses were implemented using IBM SPSS 20.

## Results

The mean values and standard deviations, skewness scores, and standard errors for the skewness of the CVLT variables at each assessment time, Wilcoxon's *z* score, Spearman's ρ, size effects and means and standard deviation of the difference T2 – T1 are shown in Table 2, for SMC, sda-MCI and mda-MCI groups.

**Table 2:** Test-retest data for the Spanish version of CVLT in the group with subjective memory complaints but without cognitive impairment (SMC), single-domain amnestic mild cognitive impairment group (sda-MCI), and multi-domain amnestic mild cognitive impairment group (mda-MCI).

**CVLT variables**	**Time 1**	**Skewness SE = 0.169**	**Time 2**	**Skewness SE = 0.169**	**Wilcoxon *z***	***rho***	***d***	**M_diff_**	**CIs (95%)**
**SMC**									
Total trial 1–5	50.57 (9.36)	0.284	51.58 (11.16)	−0.194	−2.02^*^	0.67^**^	0.10	1.02	−15.42, 17.46
Short delay free recall	10.65 (2.60)	0.104	11.07 (3.32)	−0.368	−2.30^*^	0.59^**^	0.14	0.42	−5.01, 5.85
Short delay cued recall	11.57 (2.59)	−0.334	12.02 (2.74)	−0.613	−2.60^**^	0.54^**^	0.17	0.45	−4.35, 5.25
Long delay free recall	11.40 (2.79)	−0.311	11.79 (3.26)	−0.825	−2.27^*^	0.61^**^	0.13	0.39	−4.96, 5.74
Long delay cued recall	11.84 (2.71)	−0.592	12.47 (2.90)	−0.970	−3.48^**^	0.58^**^	0.22	0.64	−4.34, 5.62
**sdaMCI**									
Total trial 1–5	32.87 (9.06)	0.095	33.89 (12.62)	−0.427	−0.61	0.70^**^	0.09	1.02	−15.17, 17.21
Short delay free recall	3.93 (1.97)	−0.178	5.30 (3.35)	−0.203	−3.31^**^	0.68^**^	0.50	1.37	−3.55, 6.29
Short delay cued recall	5.74 (2.69)	−0.334	6.91 (3.56)	−0.187	−2.74^**^	0.72^**^	0.37	1.17	−3.55, 5,89
Long delay free recall	5.22 (3.08)	−0.224	5.80 (4.05)	−0.175	−1.45	0.71^**^	0.16	0.59	−5.09, 6.27
Long delay cued recall	6.11 (2.99)	−0.360	7.13 (3.99)	−0.353	−2.45^*^	0.63^**^	0.29	1.02	−4.88, 6.92
**mdaMCI**									
Total trial 1–5	28.62 (7.30)	0.237	33.14 (10.69)	−0.226	−1.72	0.51^*^	0.49	4.52	−13.36, 22.42
Short delay free recall	3.14 (2.13)	0.173	5.10 (3.27)	−0.217	−2.96^**^	0.73^**^	0.71	1.95	−2.75, 6.65
Short delay cued recall	5.48 (2.46)	0.187	6.81 (3.16)	−0.450	−2.42^*^	0.61^**^	0.47	1.33	−3.57, 6.23
Long delay free recall	4.24 (3.32)	0.816	5.38 (3.50)	−0.267	−1.85	0.69^**^	0.33	1.14	−4.05, 6.33
Long delay cued recall	5.81 (2.60)	−0.090	7.29 (3.54)	−0.516	−2.04^*^	0.46^*^	0.48	1.48	−4.38, 7.34

*Means and Standard Deviations (in parentheses), Skewness and Standard Errors for primary measures at T1 and T2. CVLT, California Verbal Learning Test; rho, Spearman's rho; d, Cohen's d; M_diff_, difference in means; CIs, Confidence Intervals*.

* p < 0.05;

** *p < 0.01*.

Reliability coefficients for tests T1 and T2 (Spearman's ρ) were significant for all groups and ranged from 0.46 (e.g., LDCR in mda-MCI) to 0.73 (SDFR in mda-MCI).

### Practice effects

As indicated by Wilcoxon's *z* score, the SMC group obtained significant higher scores at T2 than at T1 for all variables, indicating practice effects. By contrast, both amnestic MCI groups only obtained higher scores at T2 for SDFR, SDCR and LDCR. Neither of the two groups had significant higher scores at T2 than at T1 for Total Trials 1–5 and for Long Delay Free Recall (LDFR). According to the three levels of Cohen's *d* size effect proposed by Fritz et al. (2012) (0.2 = small; 0.5 = medium; 0.8 = large), Cohen's *d* for SDFR was large in mda-MCI (0.71), medium in sda-MCI (0.50), and small in SMC group (0.14). For SDCR, the size effect was moderate in mda-MCI (0.47), less than moderate in sda-MCI (0.37), and small in SMC group (0.17). For LDCR, the size effect was moderate in mda-MCI (0.48) and small in sda-MCI (0.29) and SMC group (0.22).

**Figure 1:**
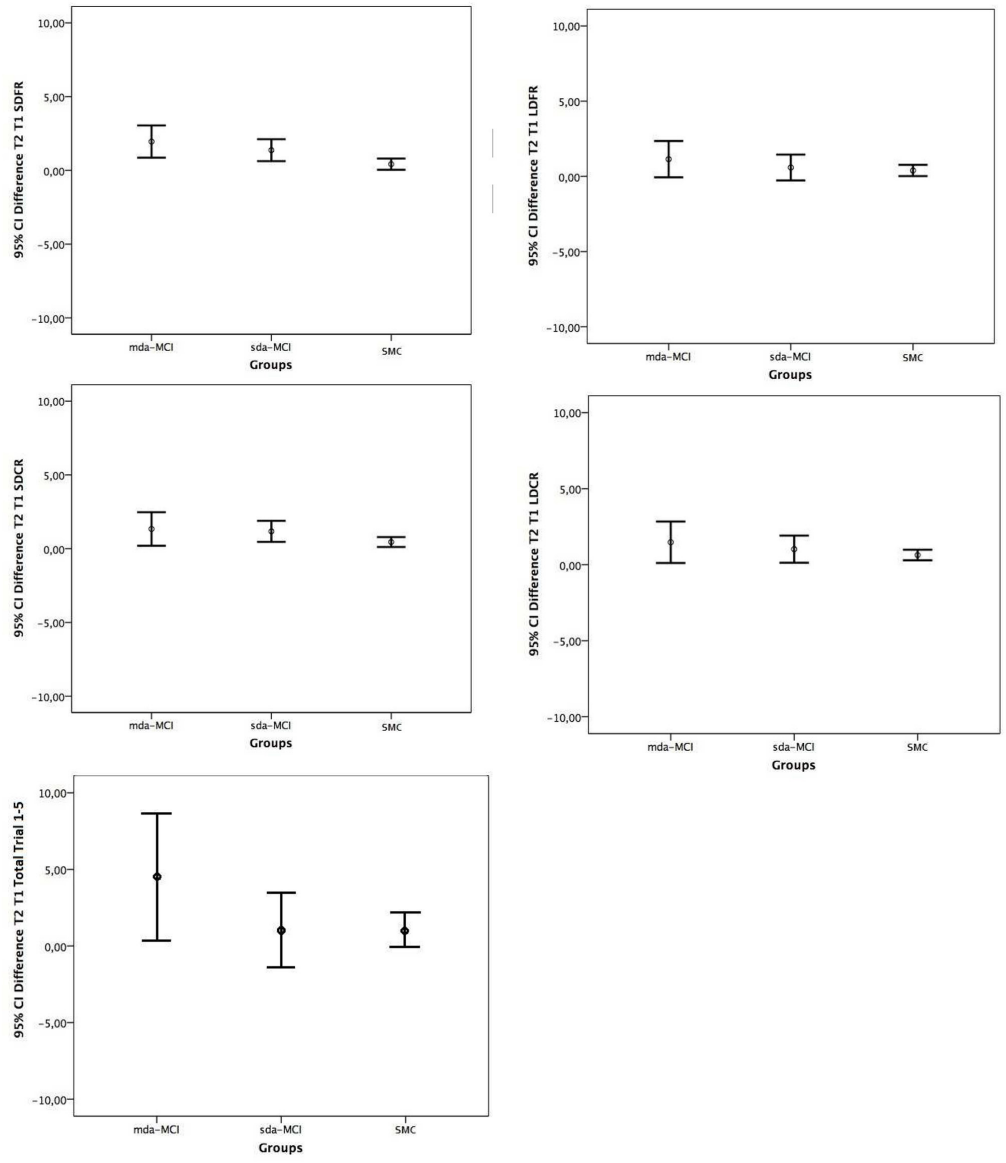
Confidence intervals (95%) for Means Difference Time 2 and Time 1 in the California Verbal Learning Test primary measures, Short Delay Free Recall (SDFR), Short Delay Cued Recall (SDCR), Long Delay Free Recall (LDFR), and Long Delay Cued Recall (LDCR) and Total Trial 1–5, for the three groups: multiple domain amnestic mild cognitive impairment (mda-MCI), single domain amnestic mild cognitive impairment (sda-MCI), and controls with subjective memory complaints (SMC).

The Confidence Intervals for differences in means, corrected by bootstrapping procedure because the different size of groups, are included in Table 2, and are graphically represented in Figure 1 for the main variables. The CIs for all variables were largest in the mda-MCI group and in the SMC group. In general, the size of CIs followed the order mda-MCI > sda-MCI > SMC, where the variability in the data increases with the CI, and having the mda-MCI group the greater variability.

### Meaningful changes

The SRB parameters from the equations predicting follow-up performance in the SMC group are shown in Table 3. Baseline scores were the strongest predictors of follow-up performance for the three CVLT variables, accounting for 46% (Total trial 1–5), 35% (SDFR), and 36% (LDFR) of the statistical variance. Age was included in the regression equations for the three measures, but only accounted for <5% of the statistical variance. Years of education was also included in Total Trial 1–5 but accounted for a very low amount of variance (<2%). The test-retest interval variable only met statistical criteria for inclusion in regression equations for Total Trial 1–5 accounting for <2% of the variance. The inclusion of age in the regression equations predicting follow-up performance explains that the percentages of stable, declined, and improved do not present the typical pattern of 90, 5, and 5% that would correspond if the baseline score was only considered.

**Table 3:** Regression coefficients and indices of significance for equations predicting follow-up scores for each of the three CVLT primary variables that showed homogeneous distribution in the group with subjective memory complaints but without cognitive impairment.

**CVLT variables**	***F*^a^**	***C*^b^**	***SEest*^c^**	**β^d^**	**β^e^**	**β^f^**	**β^g^**	***r*^h^**
Total trial 1–5	52.94	37.62	7.11	0.75	−0.25	−0.26	−0.27	0.68
Short delay free recall	30.86	9.92	2.17	0.67	−0.07			0.59
Long delay free recall	34.98	11.65	2.11	0.61	−0.09			0.60

a *All p < 0.001*.

b *Constant*.

c *Standard error of the estimate*.

d *Unstandardized Beta (slope) for baseline measure*.

e *Unstandardized Beta (slope) for age*.

f *Unstandardized Beta (slope) for education*.

g *Unstandardized Beta (slope) for test-retest interval*.

h *Correlation*.

The cross-table results comparing the meaningful change for the three groups in the three measures are shown in Table 4. There was significant (chi-square) and weak association (Cramer's *V* < 0.3) between the groups and the categories of change (stable, decline, and improvement) for the measures Total Trial 1–5 and LDFR. There were no significant differences between groups in SDFR. For Total Trial 1–5, the sda-MCI group included a lower proportion of individuals whose performance remained stable (73.9%, *CSR* = −2.5) and a higher proportion of individuals whose performance declined (17.4%, *CSR* = 2.9) than in the SMC group (87.9%, *CRS* = 1.8; and 5.8%, *CRS* = −1.7, respectively). In SDFR, the only significant difference was in decline, which was greater for the sda-MCI group (37%, *CSR* = 2.4) than for SMC group (19.8%, *CSR* = −2.4). In LDFR, the percentage of individuals whose performance declined was also higher in the mda-MCI (38%, *CSR* = 4.1) and sda-MCI groups (38.1%, *CSR* = 1.8) than in the SMC group (15.9%, *CSR* = −4.6). The percentage of individuals whose performance remained stable was lower in sda-MCI (54.3%, *CSR* = −3.5) than in SMC group (80.7%, *CSR* = 3.9). No differences between groups were found in the percentage of individuals who improve in any of the measures.

**Table 4:** Comparison of the meaningful change for the multiple domain amnestic mild cognitive impairment (mda-MCI), single domain amnestic mild cognitive impairment (sda-MCI), and group with subjective memory complaints but without cognitive impairment (SMC) in the three studied primary measures.

**CVLT variables**		***χ^2^ (df,N)***	***V***	**mda-MCI**		**sda-MCI**		**SMC**		**Groups comparison**
				***% (n)***	***CSR***	***% (n)***	***CSR***	***% (n)***	***CSR***	
Total trial 1–5		10.05^*^ (4,274)	0.135							
	Stable			90.5 (19)_a,b_	0.6	73.9 (34)_b_	−2.5	87.9 (182)_a_	1.8	sda-MCI < SMC
	Decline			0 (0)_a,b_	−1.3	17.4 (8)_b_	2.9	5.8 (12)_a_	−1.7	sda-MCI > SMC
	Improve			9.5 (2)	0.5	8.7 (4)	0.5	6.3 (13)	−0.7	
Short delay free recall		7.75 (4,274)	0.119							
	Stable			71.4 (15)	−0.4	63.0 (29)	−2.0	77.8 (161)	2.0	
	Decline			28.6 (6)_a,b_	0.6	37.0 (17)_b_	2.4	19.8 (41)_a_	−2.4	sda-MCI > SMC
	Improve			0 (0)	−0.7	0 (0)	−1.0	2.4 (5)	1.3	
Long delay free recall		23.35^***^ (4,274)	0.206							
	Stable			61.9 (13)_a,b_	−1.4	54.3 (25)_b_	−3.5	80.7 (167)_a_	3.9	sda-MCI < SMC
	Decline			38.1 (8)_a_	1.8	45.7 (21)_a_	4.1	15.9 (33)_b_	−4.6	mda-MCI, sda-MCI > SMC
	Improve			0 (0)	−0.8	0 (0)	−1.2	3.4 (7)	1.5	

*V, V de Cramer; CSR, corrected standardized residuals*.

* p < 0.05;

*** p < 0.001. Subscripts

a,b *indicate significant differences from paired comparisons between proportions (columns/groups) of individuals whose performance remained stable, declined or improved for each of the CVLT measures (corrected standardized residuals, p < 0.05). Different subscript indicate significant differences between pairs*.

## Discussion

The Spanish version of the CVLT was used to measure changes in verbal learning and memory after 1 year and half interval in three different groups of participants, with the aim of identifying variables that might affect performance at follow-up, patterns of change related to practice effects, and the proportion of individuals whose performance improves, declines, or remains stable. The study supports the mid-term test-retest reliability of the CVLT in two samples of patients with aMCI and in a SMC group. Test-retest stability coefficients were generally adequate. These findings were consistent with those of studies in which the CVLT was administered with shorter test-retest intervals to healthy adults (Woods et al., 2006) and to other clinical samples (see Tröster et al., 2007, for 17-month retest interval used with Parkinson Disease patients). The data provide new information regarding the applicability of the CVLT in a follow-up evaluation of memory for clinical and research purposes.

The results show that performance on the CVLT primary measures SDFR, SDCR, and LDCR improved, at a group level, in both types of aMCI patients and in the SMC group over the 18-month interval. However, improvement on Total Trial 1–5 (total number of words recalled on the five trials) and Long Delay Free Recall (total number of words recalled after a long delay and without semantic cues) only was significant in the control group (SMC). These results may indicate the existence of practice effects in some recalling variables for all participants due to repeated measures using the same instruments according to Knight et al. (2007), Rabbit et al. (2004), and Salthouse et al. (2004), and the lack of that practice effect on the most memory-demanding variables for the two amnestic MCI groups. In this regard, our data seem to indicate that the ability to benefit from previous exposure to the same instrument in a year and half interval may be present in both cognitively healthy individuals and in aMCI patients (regardless of the number of domains affected) except for the most memory-demanding variables such as Long Delay Free Recall in which only the control individuals take advantage. These results indicate that implicit memory involved in practice effect is preserved in aMCI patients as in normal aging (Swick and Knight, 1997) whereas explicit memory more involved in Long Delay Free Recall seems to be deteriorate in aMCI patients (Brown et al., 2009). Age, years of education and test-retest interval only accounted for a small amount of the variance (between 2 and 5%) when entered as predictors in the SRB procedure. These results provide evidence to understand the practice effect as a universal phenomenon, which occurs in normal aging, MCI and dementia diagnoses, even in very old ages (Dodge et al., 2011; Balasubramanian et al., 2012; Machulda et al., 2013), independently of the number of cognitive domains affected, and the type of instrument used (Calamia et al., 2012).

Although, the size effect of practice effect for some variables was greater in the aMCI groups than in the SMC group at follow-up, to achieve the same confidence level, aMCI groups need larger range due to bigger variability. The increased variability in the change scores for the aMCI groups, especially for the mda-MCI group, is consistent with the notion of instability of the MCI as a diagnostic and clinical entity (Han et al., 2012; Facal et al., 2014). The CIs for all variables followed the order mda-MCI > sda-MCI > SMC, with the variability in the data increasing with the CI. The mda-MCI group shows the greater variability and, accordingly, the highest proportion of change. In this respect, the data on the mda-MCI group was the most variable (large CIs), which may explain the high mean value of practice effects at follow-up but also the large proportion of participants whose performance in relation to learning variables declined.

Although, practice effects are evident in both aMCI groups, it is not clear whether this increase represents meaningful change. The SRB approach showed that the improved performance occurred in a very low proportion of participants, with no significant differences between the aMCI group and the SMC group. However, the proportion of individuals whose performance declined was higher for the single-domain aMCI group in all the measures and for the multiple-domain in LDFR than for SMC group, which remained the most stable. A higher proportion of participants whose performance on these variables declined over time indicate a higher proportion of participants whose learning capacities are impaired and will become more forgetful over time. A similar trend has already been observed in a previous cross-sectional study carried out by Greenaway et al. (2006), in which MCI participants displayed a lower learning capacity and more rapid forgetting (among other indices) than SMC group, but performed better than individuals with AD. The data do not support a previously reported pattern of impairment starting on immediate recall and subsequently in delayed recall (Bilgel et al., 2014). Our findings show that impairments seem to appear simultaneously in short and delayed recall in both subtypes of aMCI within a period of 1 year and a half.

The RBS was calculated to obtain a predicted follow-up score in the three groups based on initial performance of SMC group and to determine the proportion of individuals in each group whose performance remained stable, declined, or improved. This approach allows other variables to be used as predictors of retest scores and to control for practice effects that can alter the final results (Calamia et al., 2012). In our study, baseline scoring was the best predictive variable for the follow-up scores; age also predicted CVLT primary measures, explaining only <5% of the variance; and years of education and time interval between retest explained only <2% of the variance. The RBS approach allows more reliable follow-up results to be obtained to study the progress of each group, since not all statistically significant effects are clinically meaningful (Peters and Katz, 2015), using relatively short retest intervals that are frequent in neuropsychological assessments.

In conclusion, this study provides evidence that patients with multiple or single domain aMCI benefit from practice when their memory is reassessed at follow-up. However, when the RBS approach is used to control for practice effects, both types of aMCI displayed a high proportion of decline in short and long delay recall, suggesting that memory impairment appears simultaneously in both recall conditions. These results support the idea previously started by Brown et al. (2009) that, whereas explicit memory tends to deteriorate in higher proportion in MCI participants, implicit memory can remain stable in both MCI and in participants with subjective memory complaints without cognitive impairment, favouring the appearance of practice effects due to implicit learning. Some limitations must be indicated. First, sizes of the sample groups are unequal, having a great difference in number of participants between MCI groups and SMC group. However, previous research in MCI and practice effects used similar diagnostic sample sizes (Schrijnemaekers et al., 2007; Brambati et al., 2009) and certain statistical procedures have been adopted to maximize the accuracy of comparisons (non-parametric tests, bootstrap to compute confidence intervals). Second, no alternative versions of the Spanish version of the CVLT were used at follow-up. In this case, examinee would learn the specific words and remember them in consecutive evaluations, and consequently larger practice effects are expected. Even if retest period are quite large, analysis with parallel versions should be used in future research. For the reference group, we have taken the group of patients who attended primary health care centres with memory complaints. Even when they do not presented cognitive impairment, this group cannot be considered a fully healthy group in their cognitive aging process (Jessen et al., 2014). Finally, lack of biomarkers is a limitation according to current research criteria for MCI diagnosis, due to the possibility of having undetected preclinical AD subjects. Replications of these studies are currently being conducted including alternative version of the Spanish test, study of biomarkers and healthy participants without memory complaints. Further longitudinal studies with more than one follow-up assessment are required to confirm the pattern of meaningful changes in learning and memory in aMCI.

## Author contributions

MC, DF, CL, AP, and OJ designed the study, conducted research, analysed data, contributed to the wrote of the manuscript and revised and approved the final version.

### Conflict of interest statement

The authors declare that the research was conducted in the absence of any commercial or financial relationships that could be construed as a potential conflict of interest.
